# Euthanasia debates in low- and middle-income countries: A narrative reflection from Brazil

**DOI:** 10.1017/S1478951525101533

**Published:** 2026-01-08

**Authors:** Fernanda Nunes de Arruda

**Affiliations:** Internal Medicine, Hospital das Clínicas da Faculdade de Medicina da Universidade de São Paulo (USP), São Paulo, Brazil

Recently, while driving to my postgraduate course in palliative care, I opened *Café da Manhã*, a widely accessed Brazilian news podcast, and coincidentally the featured topic was “The Debate on Dignified Death in Brazil.” The episode began with interviews of representatives from a recently established civil association composed of citizens, lawyers, health professionals, and communicators advocating for the legalization of physician-assisted suicide under the slogan “Eu Decido” (“I Decide”). Throughout the 30-minute program, the concept of a “dignified death” was repeatedly equated with euthanasia and assisted dying. Although palliative care was briefly mentioned, the central narrative revolved around the individual right to choose death.

I arrived at class with a sense of discomfort. In a country where millions still face delayed diagnoses, limited access to essential medications, and fragmented end-of-life care, the public discourse on dying with dignity was being framed almost exclusively around assisted dying – arguably the least urgent component of our broader struggle to promote dignity at the end of life. The reduction of a multifaceted issue to a single, highly polarized solution felt deeply out of step with the needs of patients within our health system.

Debates about euthanasia are, by nature, complex. Throughout my clinical experience in Brazil’s public health system (Sistema Único de Saúde, SUS), my own views have shifted as I confronted these issues in practice. At times, the idea of choosing to end intolerable suffering appeared ethically coherent. At other moments, the decision seemed inseparable from structural inequities and what Latin American bioethics names *mistanásia* – death caused not only by disease but by abandonment, neglect, and failure of the health system. When asked recently about my personal stance on the legalization of euthanasia, I realized that a responsible answer required reflection and attention to the sociopolitical context of low- and middle-income countries (LMICs). This narrative emerges from that effort.

## Autonomy and its limits in contexts of inequality

Proponents of euthanasia frequently emphasize the centrality of individual autonomy in end-of-life decisions. Autonomy is indeed one of the most valued principles in health ethics, particularly in societies that emphasize individual liberties. However, scholars from McGill University have argued that granting absolute precedence to autonomy may compromise competing values, protections, and social responsibilities. Their perspective highlights that individual decisions do not exist in a vacuum; they reverberate through families, communities, and the medical profession itself. According to the authors, prioritizing autonomy without considering its broader consequences risks undermining essential safeguards embedded in democratic and professional institutions (Gerson et al. 2015).

Other scholars, however, emphasize that autonomy is not merely a political construct but an existential one. They argue that situations of intense and refractory suffering may threaten a patient’s sense of identity and dignity, prompting requests for assisted dying in ways that cannot be reduced to social determinants alone. They also note that in Switzerland – one of the countries where assisted suicide is legal – the steady rise in assisted deaths has not been accompanied by an increase in total suicide rates, suggesting no evidence of a “runaway” effect or loss of public control (Biller-Andorno 2020).

These contrasting viewpoints illuminate an essential tension: while autonomy is indispensable, it is never exercised independently of structural conditions. This tension becomes sharper in LMICs, where social vulnerability, uneven healthcare access, and delayed diagnoses profoundly shape the experience of suffering.

## International position statements: Palliative care before assisted dying

An influential contribution to this debate comes from the position statements of the International Association for Hospice and Palliative Care (IAHPC) and the European Association for Palliative Care (EAPC). Both organizations oppose the legalization of euthanasia and assisted suicide until universal access to palliative care and essential medications is guaranteed (European Association for Palliative Care (EAPC) 2016; International Association for Hospice and Palliative Care (IAHPC) 2017).

The IAHPC frames the issue not as an ethical condemnation of euthanasia itself but as a necessary safeguard against using assisted dying as a substitute for inadequate palliative care. The organization warns that in countries with limited resources, euthanasia may be perceived as a “solution” to suffering caused by insufficient investment in healthcare systems.

Similarly, the EAPC affirms that euthanasia is not part of palliative care and should not be performed by professionals within this field. It emphasizes the responsibility of societies to ensure the availability of specialist palliative care services, adequate financing, education, and research. Only after ensuring equitable access to symptom management and psychosocial support can a society responsibly debate whether assisted dying aligns with its ethical and cultural values.

These statements carry particular weight in LMICs, where restrictive opioid regulations, underfunded health systems, and limited training in palliative care leave millions without access to adequate symptom control. In such contexts, the risk is that requests for assisted dying may reflect a lack of alternatives rather than a genuine expression of autonomous choice.

## The Brazilian landscape: When suffering is systemic

From my work within the SUS, I recall numerous patients whose long trajectories were marked by suffering that could have been prevented or alleviated with timely care. Delayed diagnoses, fragmented referrals, and poor access to analgesia, psychological care, and home support often resulted in patients arriving at tertiary services with overwhelming physical and emotional distress. In these encounters, some expressed a desire for death – not out of philosophical reflection on autonomy but due to exhaustion and abandonment.

In such cases, the appeal of assisted dying cannot be understood separately from the context of *mistanásia*. To legalize euthanasia in countries with profound social inequities risks legitimizing a “quick solution” to suffering that, in many instances, is produced or exacerbated by the failures of the health system.

This concern is not hypothetical. In under-resourced systems, euthanasia could be interpreted – explicitly or implicitly – as a cost-effective strategy to address overcrowding, scarcity of hospital beds, and strained budgets. Equally concerning is the potential misuse of palliative care itself, particularly if implemented without sufficient training or if viewed as a “cheaper alternative” rather than a comprehensive approach prioritizing quality of life. These possibilities underscore the importance of robust palliative care systems before any legislative changes are considered.

## Reframing “dignified death” in LMIC contexts

Public discussions on dignified death in LMICs should begin not with assisted dying but with the structural determinants that shape who suffers, who receives care, and who is abandoned. Before advancing debates centered on individual choice, societies like Brazil must first guarantee early diagnosis, continuity of care, symptom control, and universal access to palliative care.

Only when these foundations are established can discussions about euthanasia occur without obscuring systemic failures under the language of autonomy. In societies marked by structural inequity, “dignity” at the end of life cannot be understood merely as the right to choose death; it must be built collectively through access to healthcare, social protection, and compassionate care long before such decisions arise.

The debate on euthanasia in LMICs must therefore be contextualized within histories of inequality and ongoing struggles for equitable access to healthcare. Autonomy remains a central ethical principle, but one that is severely constrained when structural injustices are pervasive. If dignity is to guide end-of-life policy, it must be grounded in justice as well as choice.

## References

[ref1] Biller-Andorno N (2020) Debating assisted dying: autonomy and social responsibilities. *Swiss Medical Weekly* 150, w20345. doi:10.4414/smw.2020.20345 33085769

[ref2] European Association for Palliative Care (EAPC) (2016) *EAPC Position Statement on Euthanasia and Physician-Assisted Suicide*. Milan: EAPC.

[ref3] Gerson SM, Preston TA and Emanuel EJ (2015) Physician-assisted suicide. *New England Journal of Medicine* 372, 895–899. doi:10.1056/NEJMsa1412930.

[ref4] International Association for Hospice and Palliative Care (IAHPC) (2017) *Position Statement on Euthanasia and Assisted Suicide*. Houston: IAHPC. 10.1089/jpm.2016.0290 PMC5177996 27898287

